# Psychometric Properties of Translation of the Child Perception Questionnaire (CPQ_11-14_) in Telugu Speaking Indian Children

**DOI:** 10.1371/journal.pone.0149181

**Published:** 2016-03-01

**Authors:** Santhosh Kumar, Jeroen Kroon, Ratilal Lalloo, Newell W. Johnson

**Affiliations:** 1 School of Dentistry and Oral Health, Griffith University, Queensland, Australia; 2 Population and Social Health Research Programme, Menzies Health Institute Queensland, Australia; Navoadaya Dental College and Hospital, mantralayam Road, INDIA

## Abstract

Oral health related quality of life research among children in India is still nascent and no measures have been validated to date. Although CPQ_11-14_ has been previously used in studies from the Indian sub-continent, the instrument has never been tested for cross-cultural adaptability. This study aimed to assess the validity and reliability of CPQ_11-14_ in Telugu speaking Indian school children. Primary school children of Medak district, Telangana State, India, were recruited by a multi-stage probability sampling method. The translated questionnaire was initially pilot tested on a small subset of children (n = 40). Children with informed consent from parents (N = 1342) were then provided with questionnaires containing the Telugu translation of CPQ_11-14_, followed by a clinical examination conducted by a single examiner, using Basic WHO survey methods for dental caries, malocclusion, and Dean’s Fluorosis index. Children (n = 161) in randomly chosen schools were re-administered the same questionnaire after a two week interval to test reliability of CPQ_11-14_ on repeated administrations. Internal consistency and test-retest reliability as determined by Cronbach’s alpha and Intra-class correlation coefficient for overall CPQ_11-14_ scale were 0.925 and 0.923, respectively. CPQ_11-14_ discriminated between the categories of fluorosis and malocclusion while its discriminant validity with respect to dental caries was limited. CPQ_11-14_ also demonstrated good construct validity with both overall CPQ_11-14_ and its subscales having significant positive correlation with global ratings of oral health and overall wellbeing, even after adjusting for confounding variables. CPQ_11-14_ had a correlation of 0.405 with self-evaluated oral health and 0.407 with self-evaluated impact of oral health on overall wellbeing. In conclusion, Telugu translation of CPQ_11-14_ demonstrated good internal consistency and excellent reliability on repeated administrations after two weeks. It also exhibited good discriminant and construct validity.

## Introduction

Quality of Life (QoL) has been defined by the World Health Organization (WHO) as “an individual’s perception of their position in life in the context of the culture and value systems in which they live and in relation to their goals, expectations, standards and concerns” [1]. These concepts apply to Health Related Quality of Life (HRQoL) including Oral Health Related Quality of Life (OHRQoL). OHRQoL is integral to general health and wellbeing [2] and comprises several dimensions including absence of impairment, appropriate physical, emotional and social functioning [3]. It is a holistic concept which does not rely solely on traditional clinical variables [4,5], which complements clinical indicators by determining the subjective functional and psycho-social impacts of oral disease on overall wellbeing [4]. This has led to an increase in research in recent decades [6] and although most papers report on studies conducted in adult populations, OHRQoL assessment in children has gained momentum very recently [7], which could be attributed to the introduction of measures for assessment in children.

Initially it was assumed that QoL measures reported by children themselves might not yield valid responses compared to proxy instruments answered by parents or caregivers on behalf of their children [8]. Recently several age-specific and self-administered QoL instruments have been introduced which were found to be valid. For instance, the Scale of Oral Health Outcomes for children aged 5-years [9], the Child Perception Questionnaire (CPQ)_8-10_ [10] and CPQ_11-14_ [11] for children aged 8–10 and 11–14 years respectively. CPQ does also have an analogous parental child perception questionnaire [12] which can be used as a proxy to CPQ. Child-OIDP [13], Pediatric Quality of Life Inventory (PedsQL) Oral Health Scale [14] and other instruments have also been found to be valid self-administered OHRQoL instruments for children with few limitations. CPQ_11-14_ has been most widely used and has been tested for cross-cultural adaptation in many countries including Thailand [15], Cambodia [16], Korea [17], Peru [18], Brazil [19, 20], China [21], Australia [22], New Zealand [23], UK [24], Canada [11], Saudi Arabia [25], Germany [26], Denmark [27] and Italy [28].

OHRQoL research among children in India is still nascent and no measures have been validated to date. Although, CPQ_11-14_ has been previously used in studies from the Indian sub-continent [29], the instrument has never been tested for cross-cultural adaptability. Telugu is the third most spoken language in India, estimated to be used by 74 million individuals, principally in the States of Telangana and Andhra Pradesh [30]. This study aimed to assess the validity and reliability of CPQ_11-14_ in Telugu speaking school children in Telangana.

## Methods

### Study population and sampling procedure

This validation study is part of a research project that aims to evaluate the effect of various socio-demographic and parent related variables on oral hygiene behaviour, oral health and OHRQoL of children. Ethical approval has been obtained from the Griffith University Human Research Ethics Committee (Ref No: DOH/12/14/HREC) and the ethics committee of Panineeya Dental College, India (Ref No: 00126). The study sample was recruited by a multi-stage probability sampling method from Medak District. Ten of 46 subdistricts were randomly selected. At the second stage schools were randomly selected proportional to the total number of schools in each subdistrict. Children in the 6^th^ grade were invited to participate from each chosen school. Permissions were also obtained from the District Educational Office and individual school authorities. Written informed consent was obtained from parents approving each child’s participation.

Children were visited in their schools and those willing to participate were initially provided with a booklet containing information sheet, parent questionnaire (comprised questions on Socio-economic status, family environment, health related behaviour, parent perception of child’s OHRQoL and its impact on family, parent child relationship and parenting style) and consent forms. On a subsequent visit, child questionnaires (comprised questions on health related behaviour, CPQ_11-14_ and parent-child relationship) were administered and clinical examinations conducted on those children whose parents returned signed consent forms. All participants were provided with oral hygiene and treatment advice along with an oral hygiene kit consisting of tooth paste (Colgate® dental cream) and a toothbrush (Colgate® Zigzag). In order to evaluate test-retest reliability, questionnaires were readministered to children in four randomly chosen schools. Questionnaires were considered to be invalid if more than half of the questions in each subscale were unfilled.

### Translation and cross-cultural adaptation of Telugu CPQ_11-14_

A Universalist approach was followed [31] aiming to achieve all six types of equivalence. The procedure of translation and cross-cultural adaptation followed guidelines proposed by Beaton et al., [32]. At first, two independent translators (one of them being the principal investigator, SK) conducted forward translation of the CPQ_11-14_ from the original English version to Telugu. This was followed by back translation of the Telugu version, which was synthesised with consensus of both the forward translators into English, by two independent translators. A consolidated version was then developed by an expert committee that comprised all the translators, a public health dentist and two school teachers who were postgraduates in English and Telugu literature respectively. This version was pilot tested by administering it to 40 children in one school, all of whom were later interviewed by one of the authors (SK) to identify any difficulties in understanding. Minor modifications were made at this stage based on the input from the children and a final version of Telugu CPQ_11-14_ developed (S1 File). This consisted of 37 items categorized under four domains (Oral symptoms, Functional limitations, Emotional wellbeing and Social wellbeing). The response for each item ranged from a score of 0 to 4 on a Likert scale of “not at all” to “almost every day”. A further two items on the overall self-rating of oral health and its effect on overall wellbeing were included with Likert scale responses “Excellent” = 0 to “Poor” = 4 and from “Not at all” = 0 to “Very much” = 4 respectively.

### Clinical examination

Clinical examinations were performed under natural day light by a single examiner (SK) who recorded dental caries experience, fluorosis and malocclusion. Dental caries assessment in the permanent dentition was recorded by DMFT [33]: a tooth was diagnosed as carious when a lesion in a pit or fissure, or on a smooth surface, had an unmistakable cavity, undermined enamel, or detectably softened floor or wall [34]. Dean’s fluorosis index [35] was scored from 0 to 4 (0 is given for no fluorosis and 4 for severe fluorosis characterized by confluent pitting, corroded appearance and brown stains spread over most tooth surfaces). A subject was considered to have malocclusion when he/she had any kind of malocclusion classified as Class I/II/ III by Angle [36], oligodontia or gross orthodontic problems which needed orthodontic treatment.

### Statistical analysis

Statistical analysis was performed using SPSS 22.0 (IBM, New York). Internal consistency of the domains and overall CPQ_11-14_ were assessed by Cronbach’s alpha. The test-retest reliability on repeated administrations was evaluated by Intra-class Correlation Coefficient (ICC) two-way mixed consistency method. An ICC of <0.4 is considered to be poor, 0.41–0.60 as moderate, 0.61–0.80 as good and >0.8 as excellent [37]. Normality test using Kolmogorov-Smirnov Test showed that the subscale and overall CPQ_11-14_ data in relation to various dependent variables deviated significantly from normality, hence non-parametric tests were used. Discriminant and construct validity were assessed by comparing the summary scales between the clinical diagnosis categories and global ratings respectively. Kruskal Wallis H test was used to assess the statistical differences in summary scores between the categories of caries severity and global ratings while the Mann Whitney U test was used for malocclusion and fluorosis. In addition, Spearman Correlation was used to assess the relationship between the summary scales and global ratings. As the correlation between the global ratings and summary scales could be confounded by other variables, partial ‘r’ was calculated by adjusting for gender, socio-economic status and clinical diagnosis.

## Results

Questionnaires were distributed to 1580 individuals, of which 1342 were returned, a response rate of 85%. There were no incomplete questionnaires. The final study sample thus consisted of these 1342 school children, aged 11–14 years (Mean±SD: 12.17±0.80), of which 59% were boys. For test-retest reliability, questionnaires were readministered to 180 subjects, of which 161 children returned complete questionnaires.

Internal consistency for the overall CPQ_11-14_ as determined by Cronbach’s alpha was 0.907, regarded as excellent. Emotional Wellbeing (EWB) and Social Wellbeing (SWB) subscales had Cronbach’s alpha of >0.8, while the internal consistency of the other two subscales was below 0.8. The test-retest reliability of all the subscales and overall scale was excellent with ICC’s of >0.8 (**Table 1**). Functional limitation domain had the least (0.805), while overall CPQ had the highest ICC (0.923).

**Table 1:** Internal consistency, test-retest reliability and descriptive data of overall OHRQoL and its subscales.

	Number of items	Mean	ICC	95% CI	Cronbachs alpha
**Oral symptoms**	6	4.87±3.54	0.863	0.818–0.898	0.624
**Functional limitations**	9	4.66±4.37	0.805	0.743–0.853	0.723
**Emotional wellbeing(EWB)**	9	4.47±5.74	0.876	0.835–0.908	0.869
**Social wellbeing(SWB)**	13	3.13±5.04	0.921	0.894–0.942	0.845
**CPQ**_**11-14**_	37	17.15±15.75	0.923	0.896–0.943	0.907

Approximately, 70% were caries free and severe caries (DMFT>3) was only found in 7% of the subjects. The prevalence of malocclusion and moderate to severe grades of fluorosis was 16.6% and 4.9% respectively. Very few subjects (1.04%) had decayed teeth with pulpal involvement (*not presented in tables*). **Table 2** demonstrates that children with malocclusion had significantly higher scores in EWB domain and for overall CPQ_11-14_ when compared to those with no malocclusion. Children with moderate to severe forms of fluorosis had poorer scores in all the OHRQoL domains than those with no to mild fluorosis. However, there was no discrimination in OHRQoL scores between the children belonging to various dental caries severity categories.

**Table 2:** Discriminant validity: CPQ_11-14_ and its subscales in relation to dental caries, malocclusion and fluorosis severity.

	N	Oral	Functional	Emotional	Social well	OHRQoL
		symptoms	limitations	well being	being	
		Median(IQR)	Median(IQR)	Median(IQR)	Median(IQR)	Median(IQR)
**Dental caries**						
DMFT = 0	938	4 (5)	4 (6)	2 (7)	1(4)	13 (19)
DMFT = 1–3	311	4 (5)	3 (5)	2(7)	1(4)	11 (17)
DMFT>3	93	4 (6)	3 (5)	3(6.5)	1(5)	14 (17)
**Malocclusion**						
No	1119	4 (5)	3 (6)	2 (6)	1 (4)	12 (18)
Yes	223	5 (6)	4 (7)	4 (10)^‡^	2 (5)	16 (22)*
**Fluorosis**^∞^						
No to mild	1063	4 (5) ^†^	4 (6) ^†^	2 (6) ^†^	1 (3) ^†^	12 (17) ^†^
Moderate to severe	66	9.5 (6)	9.5 (7)	13 (12.3)	10 (11.5)	40 (26)

*Mann whitney U test, p<0.05

^†^Mann Whitney U test, p<0.001

^∞^ Fluorosis examination was done only in 1129 subjects

**Table 3** shows that both the subscale scores and CPQ_11-14_ final score increased as the subjective rating of oral health deteriorated from ‘excellent to poor’. With no exception, all the CPQ_11-14_ subscales and overall CPQ_11-14_ score had significant positive correlation with global ratings of oral health and wellbeing (i.e., subjects who rated their oral health and its impact on overall wellbeing as poor and very much respectively had the highest subscale and overall OHRQoL scores). The lowest correlation existed between functional limitation and global ratings (0.269 with self-evaluated oral health and 0.311 with self-evaluated impact of oral health on overall wellbeing) while the highest correlation was observed for total CPQ_11-14_ score (0.405 with self-evaluated oral health and 0.407 with self-evaluated impact of oral health on overall wellbeing). The correlation was found to be significant even when the correlation coefficient was adjusted for known variables like gender, SES, and clinical diagnosis categories (**Table 4**). In general, the strongest correlation was observed between the total CPQ_11-14_ score and global ratings.

**Table 3:** Construct validity: overall CPQ_11-14_ and its subscale scores in relation to global self-rating of oral health and impact of oral health on overall wellbeing.

	N	Oral symptoms	Functional	Emotional	Social well	OHRQoL
		Median(IQR)	limitations	well being	being	
			Median(IQR)	Median(IQR)	Median(IQR)	Median(IQR)
Self–evaluated oral health						
**Excellent**	151	2 (3)^‡^	3 (3)^‡^	0 (2)^‡^	0 (1)^‡^	6 (9)^‡^
**Very good**	225	3 (4)	2 (3.5)	1 (3)	0 (2)	7 (14)
**Good**	387	4 (4)	3 (4)	2 (5)	1 (3)	12(16)
**Fair**	477	5 (5)	4 (5)	4 (8)	2 (5)	17 (19)
**Poor**	102	8 (5.5)	8 (7.3)	10.5 (10.3)	8.5(10.3)	35.5 (27)
Self-evaluated impact of oral health on overall wellbeing						
**Not at all**	556	3 (3.8)^‡^	2 (3)^‡^	1 (3)^‡^	0 (2)^‡^	7 (12)^‡^
**Very little**	418	5 (5)	4 (6)	3 (7)	1 (4)	15.5 (16)
**Somewhat**	258	6 (6)	5 (7)	5 (8.3)	3 (7)	21 (24)
**A lot**	57	6 (6)	6 (9)	7 (15)	6 (12.5)	28 (35)
**Very much**	53	8 (9)	5 (9.5)	3 (13)	2 (13)	18 (42)

^‡^Kruskal Wallis H test, p<0.001

**Table 4:** Construct validity: correlation of overall CPQ_11-14_ and its subscale scores with global self-rating of oral health and the impact of oral health on overall wellbeing.

	Self-rating of oral health		Self-rating of impact of oral health on overall wellbeing	
	r	Partial r	r	Partial r
Oral symptoms	0.356**	0.342**	0.386**	0.347**
Functional limitations	0.269**	0.247**	0.311**	0.286**
Emotional well being	0.408**	0.361**	0.340**	0.292**
Social well being	0.323**	0.273**	0.323**	0.312**
Overall OHRQoL	0.405**	0.376**	0.407**	0.375**

r–Spearman correlation coefficient

Partial r–correlation coefficient adjusted for gender, Socio-economic status (SES), fluorosis, malocclusion and dental caries

** Correlation is significant at 0.01 level (2-tailed)

## Discussion

The impact of a child’s oral health on QoL and its association with overall health and wellbeing has been emphasised for more than a decade [2]. Although previously the evaluation of OHRQoL in children was made using questionnaires administered to parents as proxy, it has to be understood that the perception of children and adults on the impact of oral heath on QoL is likely to be different [38]. Furthermore, it has been shown that by using appropriate techniques it is possible to obtain valid and reliable reports of HRQoL from children themselves ^11^. This has led to development of many self-administered OHRQoL instruments for children. Among many others, CPQ_11-14_ has been most widely tested with several short versions also being available [39]. This was first developed in Canada and has been found to be valid in children with dental caries, malocclusion and with craniofacial anomalies [40]. The present study aimed to evaluate the cultural acceptability, validity and reliability of a Telugu translation of CPQ_11-14_ questionnaire in representative 11- to 13-year-old school children of Medak district. It was found to be both valid as well as reliable on repeated administrations. In order to make the questionnaire acceptable to the children, a few changes were incorporated in the translated questionnaire. For instance, children were confused with the last option on the Likert scale “Every day/almost every day” which had to be changed to “almost every day”. The statement on difficulty with using a musical instrument had to be accompanied with an additional statement in parentheses as most of the children who have never used these instruments were unsure on which option to select, therefore the statement “if you have never used the instrument please select the option ‘not at all’” was added to the original item “Had difficulty in playing musical instrument like flute, trumpet or mouth organ”. In order to attain conceptual equivalence, the phrase “felt unsure of yourself” had to be changed to “lost self-confidence” and “difficult to drink with a straw” to “faced difficulty in drinking liquids (eg. cold drinks) with a straw”. When the questionnaire was piloted, most of the children could not recall that they needed to choose option “not at all” if the impact was due to reasons other than those related to their teeth, lips and jaws. Therefore, while answering the questionnaire children were asked to read each statement in functional limitation, EWB and SWB with prefix “In the past 3 months, did you ever “statement”, if you had this difficulty due to other reasons, please select “not at all”. Reliability findings demonstrated that the test-retest reliability with an interval of 2 weeks for all the subscales and overall CPQ_11-14_ was striking. All the retest questionnaires were administered exactly after two weeks, were filled by children in their classes and were returned the same day. A time frame of 2 weeks was chosen to avoid any clinical change and recollection bias. The ICC’s observed in the present study are in accordance with those reported by the developers of the CPQ_11-14_ in their validation study^11^ and that observed in a study that validated CPQ_8-10_ and CPQ_11-14_ in Brazil [20]. However, the ICC values observed in this study were greater than those reported in the previous validation studies in other languages which could be due to the longer time intervals considered in those studies, such as a month in Arabic^25^, 3 weeks in German [26] and Brazilian Portuguese [19]. EWB and SWB subscales had Cronbach’s alpha of >0.8 while the internal consistency of the other two subscales was less than 0.8. Similar trend was observed by Jokovic et al., in their validation study of CPQ_11-14_ in Canada [11]. Malocclusion has been found to have a significant negative impact on QoL [41], our findings suggest that subjects with malocclusion reported greater impact of oral heath on their daily life activities. Malocclusion has considerable psychosocial impact which influences self-confidence, social life [42] and psychological functioning [43] and orthodontic treatment in patients with malocclusion significantly contributes to improvement in OHRQoL [44]. We have found that children with malocclusion reported greater scores in EWB and SWB but the significance in difference was observed only for EWB domain. However, dental caries experience was not related to OHRQoL scores. This might be because dental caries would not cause functional limitations and psychosocial dysfunction unless associated with pain. We have observed that only 1% of the total subjects had teeth with pulpal involvement and most of the subjects were unaware of having caries in their teeth. There have been conflicting findings from the validation studies of CPQ_11-14_ with some finding an association between dental caries and OHRQoL [17–19, 23, 25] and others not [15, 26, 28, 45]. The Telugu CPQ_11-14_ showed excellent discriminant validity by discriminating the group that had fluorosis with visible changes from the group that had no distinctive changes in the teeth. All the children with no or very mild and mild forms of fluorosis were assigned to one category while the moderate and severe forms were assigned to the other category. We believed that for dental fluorosis to have a significant impact on psychosocial aspects of quality of life, it has to present with brown stains and involving most of the tooth surfaces. Studies in the past that had very few subjects with fluorosis causing aesthetic concern have observed fluorosis to have no impact on quality of life [46] and not to cause dissatisfaction to the parents [47]. Literature suggests that mild fluorosis might not be an important psychosocial concern [48] but moderate to severe fluorosis can have a significant impact on QoL [48, 49] as was observed in this study. Global ratings are the summary indicators which are used as gold standard to assess construct validity [50]. CPQ_11-14_ demonstrated good construct validity with all its subscales and overall score increasing (ie., OHRQoL decreasing) as the subject’s global ratings of oral health worsened from ‘excellent to poor’. Furthermore, all the CPQ_11-14_ subscales and overall CPQ_11-14_ score had significant positive correlation with global ratings of oral health and wellbeing both with and without adjustment for confounding variables like gender, SES and clinical diagnosis categories. This suggests that the Telugu translation of CPQ_11-14_ succeeded in measuring what it intended to measure, i.e., construct validity. Although, the strength of correlation was moderate [51], they are greater than those reported by most of the previous validation studies of CPQ_11-14_ [17, 21, 26, 50]. The strengths of this study include the sample size which is greater than most of the previously conducted validation studies of CPQ_11-14_ and representativeness of the study sample to the whole district of Medak. Furthermore, all three important clinical variables (dental caries, malocclusion and fluorosis) that are commonly used in literature for assessing discriminant validity of CPQ_11-14_ were considered in this study. However, we have not assessed criterion validity as there is no gold standard instrument that measures OHRQoL in children. In conclusion, the Telugu translation of CPQ_11-14_ demonstrated good internal consistency and excellent reliability on repeated administrations after two weeks. It also exhibited good discriminant validity with both subscale and overall scores differing between the categories of malocclusion and fluorosis. Telugu CPQ_11-14_ and all its subscales were related to global ratings of oral health and overall wellbeing, thus also had good construct validity.

## Supporting Information

S1 File Telugu version of CPQ_11-14_. (PDF)

S2 File Dataset of “Psychometric properties of translation of the Child Perception Questionnaire (CPQ11-14) in Telugu speaking Indian children”. (XLSX)
