# Supplementary material for: Psychometric Properties of Translation of the Child Perception Questionnaire (CPQ11-14) in Telugu Speaking Indian Children
Source: PLoS One. 2016 Mar 1;11(3):e0149181. doi: 10.1371/journal.pone.0149181 (PMC4773168; doi:10.1371/journal.pone.0149181)
Supplement: S1 File — (PDF) [file pone.0149181.s001.pdf]

ఈ క్రింది ప్రశ్నలకు సంబంధించిన ఏదైన ఒక స్పందనను రౌండ్ చేయండి. గడిచిన మూడు నెలల్లో ఎంత తరుచుగా ఈ క్రింద సమస్యలను ఎదుర్కొన్నారు.

	ఎప్పుడూ లేదు	ఒకటి లేదా రెండు సార్లు	కొన్ని సార్లు	అప్పుడప్పుడు	దాదాపు ప్రతిరోజు
1. పళ్లు, పెదాలు, దవడలు లేదా నోటినొప్పి	ఎ	బి	సి	డి	ఇ
2. చిగుళ్ల నుంచి రక్తం కారడం	ఎ	బి	సి	డి	ఇ
3. నోరు పెచ్చుడం కానీ, నోటిలో పుండ్లు అవడం	ఎ	బి	సి	డి	ఇ
4. నోటి దుర్వాసన	ఎ	బి	సి	డి	ఇ
5. దంతాల మధ్య లేదా లోపల ఆహారం ఇరుక్కుపోవడం	ఎ	బి	సి	డి	ఇ
6. నోటిపై భాగంలో ఆహారం ఇరుక్కుపోవడం	ఎ	బి	సి	డి	ఇ

గడిచిన మూడు నెలల్లో ఎంత తరుచుగా పళ్లు, పెదాలు మరియు దవడల వలన క్రింద తెలిపిన సమస్యలు ఎదుర్కొన్నారు. క్రింద ఇచ్చిన సమస్యలు పంటి లేదా నోరు కారణంగా కాకుండా వేరే ఏవైనా కారణాల వలన ఎదుర్కొంటే ఎప్పుడూ లేదు (ఎ) అనే స్పందనను ఎన్నుకోవాలి.

	ఎప్పుడూ లేదు	ఒకటి లేదా రెండు సార్లు	కొన్ని సార్లు	అప్పుడప్పుడు	దాదాపు ప్రతిరోజు

7. నోటి ద్వారా శ్వాసను తీసుకోవడం	ఎ	బి	సి	డి	ఇ
8. భోజనం చేయడానికి ఇతరుల కంటే ఎక్కువ సమయం తీసుకోవడం	ఎ	బి	సి	డి	ఇ
9. నిద్రపోవడంలో కష్టం	ఎ	బి	సి	డి	ఇ
10. ఆపిల్, మొక్కజొన్న లేదా మాంసము లాంటి పదార్థాలు కొరకడం లేదా నమలడంలో కష్టం	ఎ	బి	సి	డి	ఇ
11. మీ నోటిని పెద్దగా తెరవడానికి కష్టం ఎదుర్కొన్నారా	ఎ	బి	సి	డి	ఇ
12. ఏవైనా పదాలు పలకడంలో కష్టం ఎదుర్కొన్నారా	ఎ	బి	సి	డి	ఇ
13. ఇష్టమైన పదార్థాలను తినడంలో కష్టం ఎదుర్కొన్నారా	ఎ	బి	సి	డి	ఇ
14. 'స్ట్రా'తో పానీయాలను (ఉదా: కూల్‌డ్రింక్స్) తాగడంలో కష్టం ఎదుర్కొన్నారా	ఎ	బి	సి	డి	ఇ
15. వేడి లేదా చల్లని పానీయాలు సేవించడంలో కష్టం ఎదుర్కొన్నారా	ఎ	బి	సి	డి	ఇ

మీ పళ్ళు, పెదాలు, నోరు లేదా దవడల కారణంగా క్రింద తెలిపిన ప్రత్యేక భావనలు ఎంత తరుచుగా ఎదుర్కొన్నారు.

క్రింద ఇచ్చిన భావనలు పంటి లేదా నోరు కారణంగా కాకుండా వేరే ఏమైనా కారణాల వలన ఎదుర్కొంటే

' ఎప్పుడూ లేదు ' (ఎ) అనే స్పందనను ఎన్నుకోగలరు.

	**ఎప్పుడూ లేదు**	**ఒకటి లేదా రెండు సార్లు**	**కొన్ని సార్లు**	**అప్పుడప్పుడు**	**దాదాపు ప్రతిరోజు**
**16.** విసుగు లేదా మనోవేదనకు గురయ్యారు	ఎ	బి	సి	డి	ఇ
**17.** ఆత్మవిశ్వాసాన్ని కోల్పోయారు	ఎ	బి	సి	డి	ఇ
**18.** తోటి పిల్లల ముందు సిగ్గుపడడం	ఎ	బి	సి	డి	ఇ
**19.** మిగతా వారు మీ పళ్ళు, నోరు, దవడలు, లేదా పెదాల గురించి ఏమైనా అనుకుంటున్నారని ఆందోళన చెందారు	ఎ	బి	సి	డి	ఇ
**20.** వేరే వాళ్ల లాగా అందంగా లేరని బాధపడ్డారు	ఎ	బి	సి	డి	ఇ
**21.** కలత చెందారు	ఎ	బి	సి	డి	ఇ
**22.** ఆతృత లేదా భయానికి లోనయ్యారు	ఎ	బి	సి	డి	ఇ

	ఎప్పుడూ లేదు	ఒకటి లేదా రెండు సార్లు	కొన్ని సార్లు	అప్పుడప్పుడు	ప్రతిరోజు లేదా
**23.** మిగతావారిలాగా పౌష్టికంగా లేరని బాధపడ్డారు	ఎ	బి	సి	డి	ఇ
**24.** మితగావారిలా కాకుండా భిన్నంగా వున్నారని బాధపడ్డారు	ఎ	బి	సి	డి	ఇ

పాఠశాలకు సంబంధించిన ప్రశ్నలు

మీ పళ్లు, పెదాలు, నోరు లేదా దవడకు సంబంధించిన కారణాల వలన క్రింద తెలిపిన ఏవైనా సమస్యలు ఎంత తరుచుగా ఎదుర్కొన్నారు. క్రింద ఇచ్చిన సమస్యలు పంటి లేదా నోరు కారణంగా కాకుండా వేరే ఏవైనా ఇతర కారణాల వలన ఎదుర్కొంటే ' ఎప్పుడూ లేదు ' (ఎ) అనే స్పందనను ఎన్నుకోగలరు.

	ఎప్పుడూ లేదు	ఒకటి లేదా రెండు సార్లు	కొన్ని సార్లు	అప్పుడప్పుడు	దాదాపు ప్రతిరోజు
**25.** పంటినొప్పి లేదా చికిత్స కారణంగా పాఠశాలకు సెలవు పెట్టాల్సి వచ్చింది	ఎ	బి	సి	డి	ఇ
**26.** పాఠశాలలో చదువుపై దృష్టి పెట్టడంలో సమస్యలు తలెత్తడం	ఎ	బి	సి	డి	ఇ
**27.** హెూమ్ వర్క్ చేయడంలో సమస్యలు తలెత్తడం	ఎ	బి	సి	డి	ఇ
**28.** పాఠశాలలో గట్టిగా మాట్లాడడానికి లేదా చదవడానికి సంసిద్ధత చూపకపోవడం	ఎ	బి	సి	డి	ఇ

ఖాళీ సమయాల్లో లేదా ఇతరులతో ఉన్నప్పుడు చేసే పనులకు సంబంధించిన ప్రశ్నలు

క్రింద తెలిపిన అనుభవాలు పళ్లు, నోరు, పెదాలు లేదా దవడలు వలన ఎంత తరుచుగా ఎదుర్కొన్నారు. క్రింద ఇచ్చిన సమస్యలు పంటి లేదా నోరు కారణంగా కాకుండా వేరే ఏవైనా కారణాల వల్ల ఎదుర్కొంటే ' ఎప్పుడూ లేదు ' (ఎ) అనే స్పందనను ఎన్నుకోగలరు.

	ఎప్పుడూ లేదు	ఒకటి లేదా రెండు సార్లు	కొన్ని సార్లు	అప్పుడప్పుడు	దాదాపు ప్రతిరోజు
**29.** క్రీడా, సాంస్కృతిక మరియు విహార యాత్ర వంటి కార్యక్రమాలకు దూరంగా ఉండడం	ఎ	బి	సి	డి	ఇ
**30.** ఇతర పిల్లలతో మాట్లాడడానికి ఇష్టపడక పోవడం	ఎ	బి	సి	డి	ఇ
**31.** ఇతర పిల్లల ముందు నవ్వడానికి ఇష్టత చూపకపోవడం	ఎ	బి	సి	డి	ఇ
**32.** ఫ్లూట్ (లేదా పీకలాంటి) మ్యూజికల్ పరికరాలను ఉపయోగించడంలో ఇబ్బందులు ఎదుర్కోవడం (ఇలాంటి పరికరాలను ఎప్పుడూ ఉపయోగించకపోతే 'ఎప్పుడూ లేదు' (ఎ) అనే స్పందనను ఎన్నుకోగ్నలరు	ఎ	బి	సి	డి	ఇ
**33.** మిగతా వారితో సమయాన్ని గడపడానికి ఇష్టం లేకపోవడం	ఎ	బి	సి	డి	ఇ

	ఎప్పుడూ లేదు	ఒకటి లేదా రెండు సార్లు	కొన్ని సార్లు	అప్పుడప్పుడు	దాదాపు ప్రతిరోజు
**34.** ఇతర పిల్లలతో గానీ కుటుంబ సభ్యులతో గానీ వాదించడం	ఎ	బి	సి	డి	ఇ
**35.** ఇతర పిల్లలు వేరే పేర్లతో వెక్కిరించడం	ఎ	బి	సి	డి	ఇ
**36.** ఇతర పిల్లలు మిమ్మల్ని కలుపుకోకపోవడం	ఎ	బి	సి	డి	ఇ
**37.** ఇతర పిల్లలు మీ పళ్లు, పెదవులు, దవడలు లేదా నోటికి సంబంధించిన ప్రశ్నలను అడగడం	ఎ	బి	సి	డి	ఇ
